# Supplementary figures and images for: Effects of orally administered crofelemer on the incidence and severity of neratinib-induced diarrhea in female dogs
Source: PLoS One. 2024 Jan 24;19(1):e0282769. doi: 10.1371/journal.pone.0282769 (PMC10807780; doi:10.1371/journal.pone.0282769)

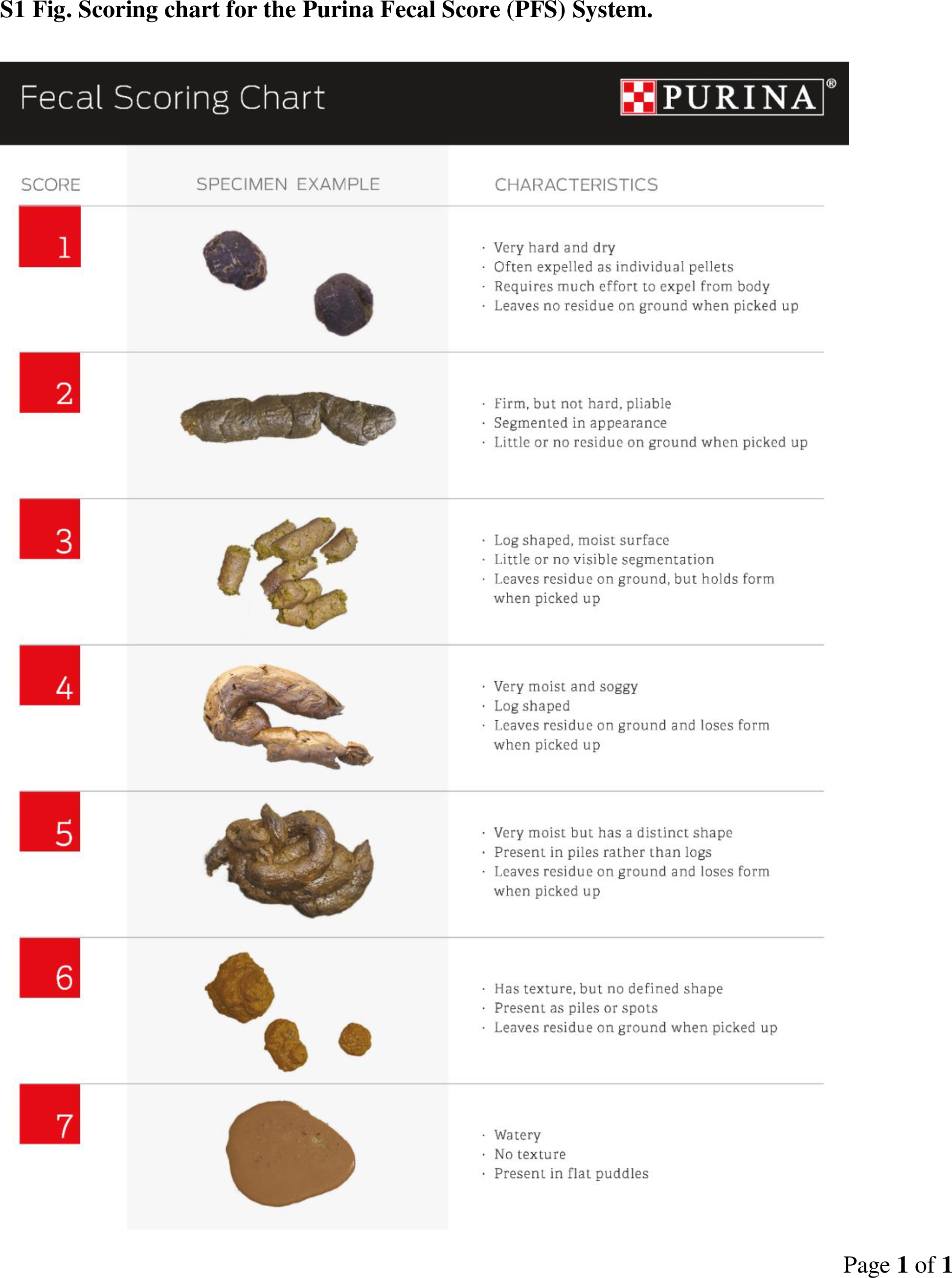

Supplement: S1 Fig — (TIF) [file pone.0282769.s001.tif]
