# Supplementary material for: Effects of orally administered crofelemer on the incidence and severity of neratinib-induced diarrhea in female dogs
Source: PLoS One. 2024 Jan 24;19(1):e0282769. doi: 10.1371/journal.pone.0282769 (PMC10807780; doi:10.1371/journal.pone.0282769)
Supplement: S1 Table — (DOCX) [file pone.0282769.s002.docx]

**S1 Table. Body weight at enrollment and body weight changes per week by treatment group over the 4-week crofelemer study period in neratinib-induced diarrhea in dogs (n=8 per treatment group).** Female beagle dogs were weighed individually daily for 28 days. The table displays body weight (kg) at enrollment as well as changes in body weight at the end of each week and the overall change in body weight from baseline (enrollment) for the 4-week study.

|  |  |  |  | P-values | |
| --- | --- | --- | --- | --- | --- |
|  | Treatment Groups | Least Square Means (LSM) | Standard Deviation (SD) | Active vs  Control | BID vs QID |
| **Body weight (kg) at enrollment** | Control | 7.30 | 0.70 | - | - |
|  | Crofelemer BID | 7.50 | 0.60 | 0.54 | - |
|  | Crofelemer QID | 6.90 | 0.20 | 0.14 | 0.02* |
| **Weekly body weight change (kg) for the combined 4 weeks** | Control | -0.37 | 0.15 | - | - |
|  | Crofelemer BID | -0.30 | 0.15 | 0.34 | - |
|  | Crofelemer QID | -0.36 | 0.15 | 0.83 | 0.49 |
| **Weekly body weight change (kg) on Week 1** | Control | -0.85 | 0.38 | - | - |
|  | Crofelemer BID | -0.77 | 0.38 | 0.68 | - |
|  | Crofelemer QID | -0.74 | 0.38 | 0.58 | 0.88 |
| **Weekly body weight change (kg) on Week 2** | Control | -0.69 | 0.37 | - | - |
|  | Crofelemer BID | -0.54 | 0.37 | 0.44 | - |
|  | Crofelemer QID | -0.66 | 0.37 | 0.89 | 0.56 |
| **Weekly body weight change (kg) on Week 3** | Control | -0.39 | 0.38 | - | - |
|  | Crofelemer BID | -0.38 | 0.38 | 0.94 | - |
|  | Crofelemer QID | -0.48 | 0.38 | 0.69 | 0.65 |
| **Weekly body weight change (kg) on Week 4** | Control | -0.58 | 0.30 | - | - |
|  | Crofelemer BID | -0.50 | 0.30 | 0.61 | - |
|  | Crofelemer QID | -0.56 | 0.30 | 0.91 | 0.72 |

Treatment groups were defined as a placebo-controlled group (CTR) receiving placebo capsules orally four times a day, crofelemer (125mg) administered orally twice daily (BID), and crofelemer (125mg) administered orally four times a day (QID) for 28 days.

* p ≤ 0.05
