# Supplementary material for: Effects of orally administered crofelemer on the incidence and severity of neratinib-induced diarrhea in female dogs
Source: PLoS One. 2024 Jan 24;19(1):e0282769. doi: 10.1371/journal.pone.0282769 (PMC10807780; doi:10.1371/journal.pone.0282769)
Supplement: S2 Table — (DOCX) [file pone.0282769.s003.docx]

**S2 Table. Food consumption per week by treatment group over the 4-week crofelemer study period in neratinib-induced diarrhea in dogs (n=8 per treatment group).** Food consumption was measured daily qualitatively. Food consumption was assigned a 3-point scale. Dogs not consuming any food during the day were assigned a point equivalent to zero, dogs consuming some of its food (equal or less than 50% of the food provided) were assigned one point, and dogs consuming most of the food provided (more than 50% of the food provided) were given two points.

|  |  |  |  | P-values | |
| --- | --- | --- | --- | --- | --- |
| **Food consumption** | Treatment Groups | Least Square Means (LSM) | Standard Deviation (SD) | Active vs Control | BID vs QID |
| **Total of 4 weeks** | Control | 31.40 | 3.93 | - | - |
|  | Crofelemer BID | 33.00 | 4.50 | 0.194 | - |
|  | Crofelemer QID | 31.30 | 2.60 | 0.526 | 0.176 |
| **Week 1** | Control | 10.60 | 2.20 | - | - |
|  | Crofelemer BID | 10.90 | 1.64 | 0.3991 | - |
|  | Crofelemer QID | 10.80 | 1.98 | 0.4491 | 0.4491 |
| **Week 2** | Control | 6.40 | 2.13 | - | - |
|  | Crofelemer BID | 7.00 | 2.62 | 0.279 | - |
|  | Crofelemer QID | 6.60 | 1.51 | 0.407 | 0.363 |
| **Week 3** | Control | 7.40 | 1.30 | - | - |
|  | Crofelemer BID | 8.10 | 1.36 | 0.137 | - |
|  | Crofelemer QID | 6.90 | 1.46 | 0.767 | 0.034 |
| **Week 4^a^** | Control | 7.00 | 0.00 | NS | - |
|  | Crofelemer BID | 7.00 | 0.00 | NS | - |
|  | Crofelemer QID | 7.00 | 0.00 | NS | NS |

^a^ All dogs in study consumed some of its food (equal or less than 50% of the food provided) were assigned one point.

Treatment groups were defined as a placebo-controlled group (CTR) receiving placebo capsules orally four times a day, crofelemer (125mg) administered orally twice daily (BID), and crofelemer (125mg) administered orally four times a day (QID) for 28 days.

* p ≤ 0.05

NS = Not Statistically Significant
