# Supplementary material for: Effects of orally administered crofelemer on the incidence and severity of neratinib-induced diarrhea in female dogs
Source: PLoS One. 2024 Jan 24;19(1):e0282769. doi: 10.1371/journal.pone.0282769 (PMC10807780; doi:10.1371/journal.pone.0282769)
Supplement: S3 Table — (DOCX) [file pone.0282769.s004.docx]

**S3 Table. Weekly average volume (mL) of subcutaneous fluid administration each week by treatment group over the 4-week crofelemer study period in neratinib-induced diarrhea in dogs (n=8 per treatment group).** Hydration status was assessed daily. Dehydration was classified as mild (minimal loss of skin turgor, semi-dry mucous membranes, normal eyes), Moderate (moderate loss of skin turgor, dry mucous membranes, weak rapid pulses, enophthalmos), and Severe (considerable loss of skin turgor, severe enophthalmos, tachycardia, extremely dry mucous membranes, weak and thready pulses, hypotension, lethargy). Cases of mild and moderate dehydration were treated with 150mL or 300mL of Lactated Ringer’s solution administered subcutaneously, respectively. Severe dehydration was not observed during the 28-day study period.

|  |  |  |  | P-values | |
| --- | --- | --- | --- | --- | --- |
| **Weekly Subcutaneous Fluid Administration (mL)** | Treatment Groups | Least Square Means (LSM) | Standard Deviation (SD) | Active vs Control | BID vs QID |
| **Total Fluids used during the 4 weeks** | Control | 28.13 | 19.2 | - | - |
|  | Crofelemer BID | 31.47 | 17.0 | 0.71 | - |
|  | Crofelemer QID | 26.79 | 16.9 | 0.88 | 0.60 |
| **Total Fluids used during Week 1** | Control | 21.43 | 16.2 | - | - |
|  | Crofelemer BID | 18.75 | 21.2 | 0.78 | - |
|  | Crofelemer QID | 21.43 | 19.8 | 1.00 | 0.78 |
| **Total Fluids used during Week 2** | Control | 61.61 | 50.5 | - | - |
|  | Crofelemer BID | 72.32 | 52.4 | 0.67 | - |
|  | Crofelemer QID | 61.61 | 47.8 | 1.00 | 0.67 |
| **Total Fluids used during Week 3** | Control | 29.47 | 37.9 | - | - |
|  | Crofelemer BID | 34.82 | 27.9 | 0.73 | - |
|  | Crofelemer QID | 21.43 | 22.9 | 0.60 | 0.39 |
| **Total Fluids used during Week 4** | Control | 0.00 | 0.0 | - | - |
|  | Crofelemer BID | 0.00 | 0.0 | 1.00 | - |
|  | Crofelemer QID | 2.68 | 7.6 | 0.23 | 0.23 |

Treatment groups were defined as a placebo-controlled group (CTR) receiving placebo capsules orally four times a day, crofelemer (125mg) administered orally twice daily (BID), and crofelemer (125mg) administered orally four times a day (QID) for 28 days.
