# Supplementary material for: Effects of orally administered crofelemer on the incidence and severity of neratinib-induced diarrhea in female dogs
Source: PLoS One. 2024 Jan 24;19(1):e0282769. doi: 10.1371/journal.pone.0282769 (PMC10807780; doi:10.1371/journal.pone.0282769)
Supplement: S4 Table — (DOCX) [file pone.0282769.s005.docx]

**S4 Table. Adverse event profile from Days 0 through 28 of 4-week crofelemer study period in neratinib-induced diarrhea in dogs (n=8 per treatment group)**. Daily clinical exams were performed during the 28-day study period. Frequency of emesis, hematochezia, lack of appetite, nausea, lethargy and sialorrhea are presented.

|  | Control | Crofelemer BID | Crofelemer QID |
| --- | --- | --- | --- |
|  | n (%) | n (%) | n (%) |
| **Emesis** | 8 (100%) | 7 (87.5%) | 8 (100%) |
| **Hematochezia** | 4 (50%) | 5 (62.5%) | 4 (50%) |
| **Lack of appetite** | 2 (25%) | 2 (25%) | 3 (37.5%) |
| **Nausea** | 2 (25%) | 3 (37.5%) | 1 (12.5%) |
| **Lethargic** | 1 (12.5%) | 2 (25%) | 2 (25%) |
| **Sialorrhea** | 1 (12.5%) | 0 (0%) | 0 (0%) |

Treatment groups were defined as a placebo-controlled group (CTR) receiving placebo capsules orally four times a day, crofelemer (125mg) administered orally twice daily (BID), and crofelemer (125mg) administered orally four times a day (QID) for 28 days.

Not significantly different between Control and the crofelemer BID or QID treatment groups.
