# Supplementary material for: Effects of orally administered crofelemer on the incidence and severity of neratinib-induced diarrhea in female dogs
Source: PLoS One. 2024 Jan 24;19(1):e0282769. doi: 10.1371/journal.pone.0282769 (PMC10807780; doi:10.1371/journal.pone.0282769)
Supplement: S5 Table — (DOCX) [file pone.0282769.s006.docx]

**S5 Table. Number of dogs with out-of-range clinical chemistry and hematology parameters on study Days 0, 7, 14, 21, and 28 by treatment group over the 4-week crofelemer study period in neratinib-induced diarrhea in dogs (n=8 per treatment group).**

|  | **Control** | **Crofelemer BID** | **Crofelemer QID** |
| --- | --- | --- | --- |
| CLINICAL CHEMISTRY |  |  |  |
| Hypoalbuminemia – Day 0 | 0 | 0 | 0 |
| Hypoalbuminemia – Day 7 | 8 | 7 | 7 |
| Hypoalbuminemia – Day 14 | 8 | 8 | 8 |
| Hypoalbuminemia – Day 21 | 8 | 5 | 7 |
| Hypoalbuminemia – Day 28 | 7 | 6 | 8 |
| Hypoproteinemia - Day 0 | 0 | 0 | 0 |
| Hypoproteinemia – Day 7 | 8 | 5 | 6 |
| Hypoproteinemia – Day 14 | 7 | 6 | 5 |
| Hypoproteinemia – Day 21 | 6 | 4 | 3 |
| Hypoproteinemia – Day 28 | 6 | 4 | 3 |
| Hypocalcemia – Day 0 | 1 | 4 | 3 |
| Hypocalcemia – Day 7 | 8 | 8 | 8 |
| Hypocalcemia – Day 14 | 8 | 8 | 8 |
| Hypocalcemia – Day 21 | 8 | 8 | 8 |
| Hypocalcemia – Day 28 | 8 | 8 | 8 |
| Elevated creatine kinase – Day 7 | 4 | 6 | 3 |
| Elevated creatine kinase – Day 28 | 5 | 0 | 3 |
| Hypoglycemia – Day 14 | 3 | 0 | 0 |
| Hypercholesterolemia – Day 14 | 3 | 0 | 0 |
| Reduced chloride – Day 14 | 3 | 0 | 0 |
| HEMATOLOGY |  |  |  |
| Increased red blood cells – Day 7 | 3 | 0 | 0 |
| Increased number of monocytes – Day 7 | 3 | 0 | 0 |
| Increased number of monocytes – Day 14 | 5 | 3 | 0 |
| Increased number of white blood cells – Day 14 | 5 | 3 | 0 |
| Increased number of white blood cells – Day 21 | 4 | 5 | 0 |
| Increased number of platelets – Day 14 | 5 | 3 | 0 |
| Increased number of platelets – Day 21 | 7 | 0 | 0 |
| Increased number of platelets – Day 28 | 3 |  |  |
| Increased number of neutrophils – Day 14 | 5 | 3 | 0 |
| Increased number of neutrophils – Day 21 | 3 | 0 | 0 |
| Increased number of eosinophils – Day 21 | 3 | 0 | 0 |
| Increased number of eosinophils – Day 28 | 5 | 0 | 0 |
| Increased percentage of reticulocytes – Day 28 | 4 | 3 | 2 |

Treatment groups were defined as a placebo-controlled group (CTR) receiving placebo capsules orally four times a day, crofelemer (125mg) administered orally twice daily (BID), and crofelemer (125mg) administered orally four times a day (QID) for 28 days.
