# Supplementary material for: Effects of orally administered crofelemer on the incidence and severity of neratinib-induced diarrhea in female dogs
Source: PLoS One. 2024 Jan 24;19(1):e0282769. doi: 10.1371/journal.pone.0282769 (PMC10807780; doi:10.1371/journal.pone.0282769)
Supplement: S6 Table — (PDF) [file pone.0282769.s007.pdf]

S6 Table. Daily raw body weights by treatment group over the 4-week crofelemer study period in neratinib-induced diarrhea in dogs (n=8 per treatment group).

Raw Body Weights (g)

Animal Number	Raw (g)																												
	Day 0	Day 1	Day 2	Day 3	Day 4	Day 5	Day 6	Day 7	Day 8	Day 9	Day 10	Day 11	Day 12	Day 13	Day 14	Day 15	Day 16	Day 17	Day 18	Day 19	Day 20	Day 21	Day 22	Day 23	Day 24	Day 25	Day 26	Day 27	Day 28
**151**	6.8	6.8	6.7	6.5	6.4	6.2	6.2	5.9	5.9	5.8	6.0	6.1	6.3	6.0	5.9	5.8	6.1	6.2	6.3	6.2	6.2	6.2	6.4	6.5	6.5	6.5	6.4	6.8	6.7
**152**	7.4	7.5	7.1	7.2	7.1	6.9	7.0	6.9	7.1	6.9	6.7	6.4	6.3	6.2	6.2	5.9	6.3	6.4	6.3	6.5	6.8	6.7	6.7	6.8	6.7	6.8	6.6	6.7	6.7
**153**	8.5	8.4	8.5	8.2	8.1	8.1	8.0	8.0	7.9	7.8	7.8	7.6	7.4	7.4	7.1	7.5	7.6	7.3	7.3	8.0	7.8	7.7	7.9	7.9	7.7	7.8	8.1	8.3	8.4
**154**	7.5	7.3	7.4	7.0	7.1	7.2	7.0	7.1	6.7	6.7	6.4	6.7	7.0	7.0	7.0	6.8	7.1	7.1	7.1	7.0	7.1	7.2	7.2	7.2	7.4	7.3	7.3	7.4	7.4
**155**	7.6	7.6	7.4	7.4	7.2	7.5	7.3	7.6	7.1	7.1	7.0	7.3	7.3	7.1	7.2	7.1	7.3	7.2	7.5	7.5	7.4	7.5	7.8	7.7	7.9	7.6	7.8	7.9	7.8
**156**	7.8	7.4	7.4	7.3	7.2	7.0	6.9	6.6	6.5	6.4	6.3	6.1	6.0	6.5	6.2	6.2	6.2	6.4	6.7	6.6	6.7	6.6	6.5	6.8	6.9	6.8	6.9	7.0	6.9
**157**	6.4	6.3	6.1	6.2	6.0	5.9	5.6	5.4	5.3	5.1	5.1	4.9	5.2	5.2	5.4	5.4	5.4	5.6	5.6	5.8	5.6	5.6	5.6	5.9	5.6	5.7	5.7	5.7	5.7
**158**	6.4	6.2	5.9	6.0	6.0	6.0	6.6	6.2	6.1	6.0	6.0	6.0	6.1	6.0	6.1	6.1	6.0	6.2	6.1	6.2	6.1	6.8	6.3	6.2	6.3	6.3	6.3	6.3	6.4
**Mean**	**7.3**	**7.2**	**7.1**	**7.0**	**6.9**	**6.9**	**6.8**	**6.7**	**6.6**	**6.5**	**6.4**	**6.4**	**6.5**	**6.4**	**6.4**	**6.4**	**6.5**	**6.6**	**6.6**	**6.7**	**6.7**	**6.8**	**6.8**	**6.9**	**6.9**	**6.9**	**6.9**	**7.0**	**7.0**
**SD**	**0.7**	**0.7**	**0.8**	**0.7**	**0.7**	**0.8**	**0.7**	**0.9**	**0.8**	**0.8**	**0.8**	**0.8**	**0.7**	**0.7**	**0.6**	**0.7**	**0.8**	**0.6**	**0.7**	**0.7**	**0.7**	**0.7**	**0.8**	**0.7**	**0.8**	**0.7**	**0.8**	**0.8**	**0.8**
**251**	6.8	6.6	6.5	6.6	6.4	6.3	6.4	6.0	6.0	5.8	5.7	5.6	5.5	5.3	5.3	5.7	5.9	6.0	5.6	5.7	5.7	6.0	5.9	5.9	5.8	5.9	5.9	5.8	6.0
**252**	6.9	6.7	6.5	6.4	6.3	6.3	6.3	6.2	6.2	6.2	6.0	6.1	6.2	6.4	6.4	6.3	6.1	5.9	6.2	6.0	6.3	6.2	6.1	6.2	6.1	6.4	6.3	6.4	6.3
**253**	6.8	6.9	6.9	6.7	6.8	6.9	6.7	6.1	6.6	6.6	6.5	6.5	6.4	6.1	6.1	6.7	6.4	6.3	6.5	6.8	6.9	6.7	6.7	6.5	6.8	6.8	6.9	6.9	6.8
**254**	7.0	6.6	6.5	6.4	6.4	6.5	6.1	6.1	6.1	5.8	5.6	5.6	5.6	5.8	5.7	5.7	5.7	5.6	5.5	5.9	5.8	5.9	6.1	5.9	6.1	6.1	6.1	6.0	6.0
**255**	7.1	7.0	7.0	6.8	6.9	6.9	6.8	6.5	6.6	6.5	6.6	6.7	6.8	6.5	6.7	6.5	6.6	6.5	6.6	6.6	6.7	6.6	6.5	6.6	6.9	6.0	6.7	6.7	6.8
**256**	6.6	6.4	6.1	6.3	6.3	6.2	6.3	6.0	5.8	5.7	5.7	6.1	6.0	6.3	6.0	6.3	6.2	6.0	6.1	6.4	6.7	6.4	6.7	6.7	6.7	6.5	6.5	6.7	6.7
**257**	6.9	6.8	6.8	6.5	6.7	7.0	6.8	6.7	6.4	6.6	6.4	6.6	6.7	6.6	6.6	6.6	6.7	6.7	6.9	6.8	6.5	6.8	6.8	7.1	6.9	6.8	6.8	7.1	6.5
**258**	7.3	7.0	7.0	6.7	6.7	6.7	6.8	6.8	6.6	6.7	6.6	6.6	6.5	6.4	6.5	6.5	6.6	6.7	6.7	6.7	6.6	6.8	6.8	6.9	6.7	6.7	6.8	7.1	6.8
**Mean**	**6.9**	**6.8**	**6.7**	**6.6**	**6.6**	**6.6**	**6.5**	**6.3**	**6.3**	**6.2**	**6.1**	**6.2**	**6.2**	**6.2**	**6.2**	**6.3**	**6.3**	**6.2**	**6.3**	**6.4**	**6.4**	**6.4**	**6.5**	**6.5**	**6.5**	**6.4**	**6.5**	**6.6**	**6.5**
**SD**	**0.2**	**0.2**	**0.3**	**0.2**	**0.2**	**0.3**	**0.3**	**0.3**	**0.3**	**0.4**	**0.4**	**0.4**	**0.5**	**0.4**	**0.5**	**0.4**	**0.4**	**0.4**	**0.5**	**0.4**	**0.4**	**0.4**	**0.4**	**0.4**	**0.4**	**0.4**	**0.4**	**0.5**	**0.3**
**351**	7.1	7.0	6.9	6.8	6.6	6.7	6.5	6.6	6.5	6.2	6.1	6.3	6.4	6.6	6.4	6.4	6.3	6.6	6.7	6.6	6.6	6.8	6.6	6.8	6.7	6.9	6.9	7.0	6.9
**352**	8.3	8.5	8.3	8.3	8.0	8.1	7.9	7.7	7.4	7.6	7.3	7.4	7.5	7.4	7.3	7.5	7.3	7.3	7.3	7.3	7.4	7.6	7.6	7.6	7.5	7.7	7.6	7.7	7.7
**353**	7.7	7.4	7.4	7.4	7.4	7.5	7.3	7.6	7.3	7.3	7.2	7.6	7.5	7.5	7.5	7.1	7.5	7.6	7.7	7.7	7.6	7.6	7.6	7.4	7.7	7.8	7.5	7.7	8.0
**354**	8.1	7.9	8.0	7.7	7.8	7.8	7.6	7.5	7.1	7.1	6.8	6.7	6.4	6.3	6.2	6.8	6.8	7.2	7.1	7.3	7.1	7.1	7.2	7.2	7.2	7.1	7.0	7.2	7.2
**355**	7.8	7.8	7.7	7.7	7.5	7.5	7.5	7.6	7.5	7.2	7.4	7.3	7.5	7.2	7.4	7.4	7.5	7.5	7.6	7.7	7.7	7.8	7.8	7.8	8.0	7.7	7.9	7.8	8.0
**356**	7.4	7.2	7.1	7.0	6.9	7.0	7.0	7.0	7.0	6.7	6.8	6.9	6.9	7.0	7.0	7.0	7.0	7.0	7.1	7.1	7.1	7.1	7.3	6.9	7.2	7.2	7.2	7.2	7.1
**357**	7.3	7.1	7.1	7.0	7.2	7.0	7.0	6.8	6.4	6.5	6.4	5.9	6.2	6.1	6.4	6.5	6.8	6.6	6.5	6.6	6.5	6.7	6.5	6.6	6.7	6.7	6.5	6.7	6.6
**358**	6.4	6.1	6.3	6.2	6.2	6.1	5.8	5.7	5.7	5.5	5.4	5.3	5.2	5.2	5.5	5.6	5.5	5.7	5.6	5.8	5.6	5.6	5.7	6.0	5.8	6.1	5.9	6.0	5.9
**Mean**	**7.5**	**7.4**	**7.4**	**7.3**	**7.2**	**7.2**	**7.1**	**7.1**	**6.9**	**6.8**	**6.7**	**6.7**	**6.7**	**6.7**	**6.7**	**6.8**	**6.8**	**6.9**	**7.0**	**7.0**	**7.0**	**7.0**	**7.0**	**7.0**	**7.1**	**7.2**	**7.1**	**7.2**	**7.2**
**SD**	**0.6**	**0.7**	**0.6**	**0.7**	**0.6**	**0.6**	**0.7**	**0.7**	**0.6**	**0.7**	**0.7**	**0.8**	**0.8**	**0.8**	**0.7**	**0.6**	**0.7**	**0.6**	**0.7**	**0.6**	**0.7**	**0.7**	**0.7**	**0.6**	**0.7**	**0.6**	**0.6**	**0.6**	**0.7**
