# Supplementary material for: Effects of orally administered crofelemer on the incidence and severity of neratinib-induced diarrhea in female dogs
Source: PLoS One. 2024 Jan 24;19(1):e0282769. doi: 10.1371/journal.pone.0282769 (PMC10807780; doi:10.1371/journal.pone.0282769)
Supplement: S8 Table — (PDF) [file pone.0282769.s009.pdf]

S8 Table. Daily Purina Fecal Scores and number of bowel movements by treatment group over the 4-week crofelemer study period in neratinib-induced diarrhea in dogs (n=8 per treatment group).

Purina Fecal Score, Day 0-7

Animal Number	Day 0	Day 0	Day 1	Day 1	Day 2	Day 2	Day 3	Day 3	Day 4	Day 4	Day 5	Day 5	Day 6	Day 6	Day 7	Day 7
**151**	2 x 2	NA	4, 5, 6, 2	NA	6 x2, 7	NA	2 x2	2 x2	2, 7	6	3, 7	6	7 x2, 6	7 x3	NA	7
**152**	2 x 2	NA	2 x2, 4	NA	5	6	1, 6	NA	5, 7 x3	2, 7	4, 7	NA	7 x3	7 x2	7 x3, 6	7
**153**	2 x2	NA	2 x2, 7	4 x2	2, 6 x2	2, 6 x2	7 x2	4	6, 7 x3	7 x3, 3	7 x4	7 x2, 5	3 x2, 4, 7	7 x2	7 x3	7, 6
**154**	2 x3	NA	2	4, 5	5 x2, 6	2 x4, 5	3, 6 x2	3, 7	4 x2	3	5 x2, 6	NA	6	7, 6	7 x2	7, 6 x2
**155**	3 x1	NA	7 x1, 6 x1, 3 x1	NA	7 x1, 3 x2	4 x1	7 x3, 5 x1	2 x1	7 x1, 6 x1	NA	3 x1, 5 x2	3 x1, 5 x1	7 x3	7 x1	7 x3	6 x1, 3 x1
**156**	3 x1	3 x1	3 x2	NA	3 x1, 7 x1	4 x1	7 x1	7 x2	7 x2	NA	7 x1	7 x2	7 x2	7 x2	7 x4	7 x1
**157**	2 x1	NA	1 x1	NA	7 x1	2 x5	NA	NA	7 x1	NA	7 x2, 3 x1	7 x1	7 x2	7 x5	7 x3	NA
**158**	2 x1	2 x1	2 x1	NA	5 x1	NA	3 x2	NA	7 x1, 6 x1	NA	3 x1, 7 x3	NA	7 x2	NA	3 x1, 4 x1	3 x1
**251**	2 x2	NA	2 x4	2 x1	1 x2	NA	1 x4, 6 x1, 7 x2	2 x1	5 x1, 6 x1, 7 x1	4 x1	4 x1, 5 x1	NA	3 x1	NA	7 x4	NA
**252**	2 x2	2 x1	2 x4	2 x1	1 x2	NA	1 x2, 2 x1	NA	3 x1, 5 x1	3 x1	4 x2	5 x1	NA	5 x1	3 x1	7 x1
**253**	3 x2	2 x1	6 x1, 1 x1, 7 x1	2 x1, 5 x1	7 x1, 2 x1, 1 x1	6 x1, 7 x2	2 x1	3 x1	3 x1, 4 x1	7 x2	3 x1, 4 x1	6 x1	1 x1, 7 x1	7 x2	7 x2	6 x1
**254**	2 x1, 3 x1	2 x1	2 x2	2 x1, 3 x1	1 x1, 2 x1	2 x2	3 x3	4 x1	3 x1, 5 x1	6 x1	5 x1, 6 x1, 7 x1	NA	7 x2	NA	7 x1, 3 x1	NA
**255**	3 x2	2 x1	7 x2, 5 x1	7 x2	7 x1	4 x1, 6 x1	6 x1, 5 x1	NA	6 x2, 7 x1	2 x1	4 x1, 6 x1	6 x1	7 x3	7 x1, 2 x1	7 x6	NA
**256**	4 x1	3 x1, 7 x1	7 x2	NA	7 x2, 3 x1	6 x1	7 x3	6 x2	7 x1, 6 x1, 5 x1	7 x2	7 x3, 6 x1	7 x2, 6 x1, 4 x1	6 x4	7 x1, 5 x1	7 x4	NA
**257**	3 x1	3 x2	NA	6 x1	5 x1	NA	7 x1, 6 x1	2 x1, 3 x1	2 x1	2 x2	3 x2	NA	7 x2	NA	7 x2	NA
**258**	3 x1, 2 x1	4 x1	1 x2	6 x1	3 x1	NA	7 x1	NA	4 x1	NA	3 x2	3 x1, 5 x1	7 x3	NA	7 x2	NA
**351**	3 x2	NA	2 x2, 3 x3	3 x2	7 x3	7 x1	2 x3, 3 x1	2 x2	2 x1, 4 x1	3 x1	3 x2	NA	2 x1, 3 x2	6 x1, 7 x1	6 x1, 5 x1, 3 x1	7 x1, 3 x1
**352**	2 x2	NA	2 x3, 3 x1	2 x1	1 x1, 6 x1, 7 x1	4 x1, 5 x1	2 x4	2 x3	NA	2 x1, 7 x1	5 x1, 7 x1	4 x1	7 x2, 4 x1	7 x3	7 x1, 3 x1	7 x2
**353**	3 x2	2 x1	NA	4 x1	1 x2	NA	2 x2	7 x1	4 x1	NA	4 x1, 5 x1	NA	3 x2, 4 x1, 7 x1	NA	4 x1	4 x1
**354**	2 x3	NA	1 x1, 6 x1	2 x1	1 x2, 7 x1	2 x2	2 x2	NA	7 x1, 3 x3	6 x1	3 x2, 6 x1	7 x1	6 x1, 7 x1	4 x1	7 x2	7 x3
**355**	2 x1	NA	3 x4	2 x1	3 x2	NA	3 x2	2 x3	4 x1	2 x1	2 x1, 4 x1	2 x1	6 x2, 5 x1	NA	7 x3, 5 x1	7 x3
**356**	3 x1	NA	4 x1	4 x1, 6 x1	7 x1	4 x1, 7 x1	7 x1, 3 x1	2 x3	7 x2, 5 x1	NA	7 x1	NA	7 x1, 5 x1, 4 x1	4 x1	7 x3, 4 x1	2 x1, 4 x1
**357**	4 x1	3 x2	3 x2	6 x1	7 x1, 5 x1	2 x1	4 x1	7 x1, 5 x1	6 x1, 3 x1	2 x1	6 x1, 4 x2	4 x2	7 x6, 6 x1	NA	7 x1	7 x1
**358**	3 x1	NA	2 x2	4 x1, 7 x1	7 x1, 4 x1	2 x2	7 x2	NA	7 x2	NA	7 x1, 6 x1	7 x2	7 x6	3 x1	7 x2	3 x1

Purina Fecal Score, Day 8-14

Animal Number	Day 8	Day 8	Day 9	Day 9	Day 10	Day 10	Day 11	Day 11	Day 12	Day 12	Day 13	Day 13	Day 14	Day 14
**151**	7	7 x2	7 x3	NA	7 x3	NA	7 x3	3	6 x2	NA	7 x3	6	7 x2	7
**152**	7, 6 x2	NA	7 x3	5	7 x2	6	7 x2	7	7	NA	7 x2	7	7 x3	7
**153**	7 x3	7 x2	7 x2	7 x2, 2 x4, 5	7 x2	7 x2, 6	7 x2	3	7 x2	7 x2	7 x3	7 x2	7 x2	7
**154**	7 x2	6	7	2, 6	7	7	7, 6	7	5 x2	6, 7	4 x2, 5, 7	2	5 x2, 6	5
**155**	7 x2, 5 x1	7 x1	7 x1, 6 x1	NA	7 x2	7 x1, 2 x2	7 x3	3 x2	7 x2	NA	7 x2, 6 x2	7 x1, 4 x1	7 x3	7 x1
**156**	7 x3	7 x1	7 x1	7 x3	7 x2	7 x2	7 x2	7 x1	7 x1	7 x1	7 x2	7 x2, 4 x1	7 x4	2 x2
**157**	7 x4	7 x1	7 x1	NA	7 x1	7 x3	7 x2	NA	7 x1, 6 x1	7 x4	7 x3	7 x1	7 x4	2 x1, 7 x1
**158**	3 x2	3 x2	4 x2	2 x2	2 x1	2 x1	2 x1	7 x1, 2 x1	7 x1, 2 x1	NA	3 x2	2 x3	4 x1, 2 x1	2 x1
**251**	7 x2	7 x3	7 x2	NA	NA	7 x2	7 x3	7 x1	7 x3	7 x2	7 x2	7 x1	7 x2	NA
**252**	NA	NA	6 x1, 7 x2	NA	7 x2	NA	NA	4 x1, 5 x1	NA	NA	NA	NA	7 x1, 2 x2	NA
**253**	7 x1	7 x1	7 x1	7 x1	7 x1	5 x1	7 x2	7 x2	7 x3	7 x1	7 x3	NA	7 x1	7 x1, 4 x1
**254**	7 x2	7 x1	7 x1	7 x2	7 x1	7 x3	NA	7 x2	7 x3	7 x1	7 x1, 3 x2	NA	3 x1	4 x1
**255**	4 x1	5 x1	7 x2	7 x2, 5 x1	7 x1	2 x1	2 x1, 3 x1	NA	2 x1	5 x2	7 x1, 5 x1	7 x2	5 x1, 2 x1	3 x2
**256**	7 x6	7 x2	7 x3	7 x3	7 x3	NA	6 x3	7 x3	7 x4	5 x1	7 x2, 6 x1, 4 x1	7 x1, 5 x1, 4 x1	7 x2, 6 x2	NA
**257**	7 x2, 5 x1	NA	4 x1	2 x1	7 x2	NA	7 x3	2 x1	7 x1, 3 x1	NA	3 x2	1 x1, 2 x2, 3 x1	2 x2	1 x1
**258**	7 x4	NA	7 x1	7 x1	7 x2	7 x1	7 x3	7 x1	7 x3	7 x2	7 x4	NA	7 x1	NA
**351**	7 x3	7 x3	7 x3	7 x1	7 x2	6 x3, 4 x1	7 x2, 6 x2	7 x1, 4 x1	7 x5	NA	6 x1, 5 x2	4 x2	7 x5	NA
**352**	NA	NA	7 x2	7 x2	7 x3	7 x1	NA	4 x1	7 x3	2 x1	7 x2, 4 x1	7 x2	7 x5	NA
**353**	6 x1	NA	7 x1	6 x1	NA	NA	4 x1	6 x1	7 x2	6 x1, 7 x1	7 x2	NA	7 x2	NA
**354**	7 x1	7 x1	7 x2	7 x4	7 x4	NA	7 x3	NA	7 x3	NA	7 x2	NA	7 x5	NA
**355**	7 x1	NA	7 x1, 6 x1	4 x2	4 x2	2 x3, 6 x1	3 x1	NA	2 x1	6 x2	7 x3	3 x1	5 x1	2 x2
**356**	7 x4, 3 x1	4 x2	7 x4	7 x1, 5 x1	4 x2	NA	2 x1, 3 x1	3 x2, 4 x1	7 x2	NA	7 x2, 5 x2	3 x1	3 x2	1 x1, 2 x2
**357**	7 x3	NA	7 x2	7 x2	7 x1	7 x2	7 x2	7 x2	7 x1	7 x3	7 x3	6 x1, 7 x1	7 x1, 6 x1	3 x2
**358**	7 x3	7 x2, 4 x1	7 x3	7 x2	7 x2	7 x1	7 x2	7 x1	7 x2	7 x1, 3 x1	7 x2	7 x2	7 x3	7 x1

Purina Fecal Score, Day 15-21

Animal Number	Day 15	Day 15	Day 16	Day 16	Day 17	Day 17	Day 18	Day 18	Day 19	Day 19	Day 20	Day 20	Day 21	Day 21
**151**	7	NA	7 x2	4	6 x1	NA	6	NA	1 x2	6	7 x2	5	7 x2	NA
**152**	7	7	7 x3	7	6 x2	7	7 x3	6	7 x2	6	6 x3	6	7 x2	2
**153**	7 x3	7, 6	7 x4, 3	7 x3	7 x2	7 x2	7 x4	NA	5	7 x3, 4	7 x3, 6	7 x2, 4	7	2 x2, 4
**154**	7, 4 x2	5	3	6 x2	4	2	2, 3	7, 6	7, 6	7	6, 5	2 x2	4, 3	2
**155**	7 x3	2 x1	7 x2, 4 x1	2 x1	3 x2	5 x1	7 x2	7 x1, 6 x1	7 x1, 3 x1	2 x1	7 x2	7 x2, 3 x1	7 x1, 3 x1	6 x1
**156**	7 x5, 6 x2	6 x1, 3 x1	7 x3	5 x1	3 x1, 4 x1	2 x1	2 x2	2 x2	7 x5	2 x1	6 x1, 3 x1	2 x1	3 x2	3 x1
**157**	7 x2, 4 x1	4 x2	7 x2	6 x1	7 x1, 5 x3	2 x2	7 x1	3 x1	3 x1	2 x1	2 x1	7 x1, 2 x1	7 x1	6 x1
**158**	2 x1	2 x2	7 x1, 4 x1	NA	7 x4	7 x1, 6 x1	7 x2	NA	3 x1	1 x1	2 x1	7 x3	7 x3	NA
**251**	6 x2	NA	4 x1	5 x1	6 x1, 7 x3	7 x2	7 x3	NA	7 x4, 5 x1	7 x1, 5 x1	NA	NA	NA	NA
**252**	7 x2	NA	7 x3	7 x1	7 x2	7 x2	7 x2	6 x2	6 x1	7 x1	NA	7 x1	NA	NA
**253**	7 x3	7 x5	7 x2	7 x2	7 x3	NA	3 x4	7 x1, 6 x1	3 x1, 4 x1	4 x2	7 x5	2 x1	7 x3	5 x1, 6 x1
**254**	4 x1	3 x1	2 x1	2 x2	7 x2	2 x1	2 x1, 7 x2	NA	7 x3, 6 x1	7 x2, 6 x1, 4 x1	6 x2, 4 x2	3 x2	6 x2, 2 x1	NA
**255**	7 x3, 4 x1	2 x2	2 x1	2 x2	2 x2	7 x1	2 x1	1 x1, 2 x1	4 x2	6 x1, 7 x1	3 x2	3 x2	7 x3	7 x1
**256**	7 x1, 4 x1, 3 x1	7 x2, 6 x1	7 x3	6 x1	7 x4	6 x1	7 x4	2 x1	7 x4, 6 x1	7 x3	7 x6	6 x1	7 x2, 6 x2	7 x2
**257**	2 x2	2 x1	2 x2	2 x1	2 x1	2 x2	NA	3 x1	5 x1, 3 x2	3 x1	7 x5, 3 x1	NA	2 x2	2 x1
**258**	4 x1	2 x1	7 x1, 2 x1	NA	NA	1 x1, 2 x1	2 x2	3 x1, 2 x1	NA	3 x1	NA	2 x1	4 x1	7 x1, 4 x1
**351**	7 x2	7 x1	7 x4	7 x3	6 x3	2 x2	4 x2	NA	2 x1, 3 x2, 5 x1	NA	3 x1, 7 x2	7 x2	7 x2, 5 x2	2 x1
**352**	7 x2	7 x3	7 x3	4 x1	7 x3, 4 x1	6 x2	7 x3	7 x3	7 x2	7 x2	7 x3	3 x1	7 x1, 4 x1	7 x1, 2 x1
**353**	5 x2	NA	NA	3 x1	NA	NA	3 x2	6 x1, 7 x1	7 x2	7 x1	6 x1, 3 x2	4 x1	3 x2	4 x1
**354**	5 x2	NA	5 x2	NA	6 x1, 4 x1, 3 x1	2 x1	3 x3	7 x1, 4 x1	7 x3	4 x2	7 x3, 6 x1	4 x2	3 x3	NA
**355**	2 x2	2 x2	4 x1	2 x1	3 x3	2 x2	3 x1, 2 x3	2 x2, 6 x1	NA	2 x1	2 x2	2 x1	2 x1	2 x1
**356**	3 x1, 2 x1	2 x1	5 x1	7 x2	7 x1, 4 x2	7 x1	7 x1, 6 x2	2 x1	2 x1	2 x1	2 x1	3 x1	7 x1, 2 x1	2 x1
**357**	5 x1, 4 x1	2 x2	3 x3	7 x1, 3 x3	7 x1	NA	7 x3, 6 x1	6 x3	7 x1, 6 x1	3 x1	7 x3, 2 x2	4 x1	6 x1, 4 x1	4 x1, 5 x1
**358**	7 x2	7 x3	6 x2, 5 x2	7 x1	7 x1, 6 x2	5 x2	7 x4	7 x1, 4 x2	7 x3, 6 x1	7 x1, 5 x1, 4 x1	3 x1	7 x1, 3 x1	3 x2	3 x1

Purina Fecal Score, Day 22-28

Animal Number	Day 22	Day 22	Day 23	Day 23	Day 24	Day 24	Day 25	Day 25	Day 26	Day 26	Day 27	Day 27	Day 28
**151**	7 x2	NA	3	NA	7, 5, 6	7	2	NA	7 x3	1	1, 5	1	6
**152**	7, 4 x2	2	4, 6, 7 x2	NA	1	2	2	NA	7 x2, 6	7 x2	2, 7 x3	6	7 x4
**153**	7 x3	2 x2	2 x3, 1	2	7 x3	2, 7 x2	7 x4	3	2, 3 x3, 6	1	7 x2, 6 x3	5 x2	7 x5
**154**	4, 5	2, 7	1 x3	NA	2	5, 6	7 x3, 6	NA	2, 5, 6	1, 2	2 x2	2 x2	3
**155**	7 x2, 5 x1	6 x1, 5 x1, 4 x1	7 x2, 4 x1	7 x1, 5 x1	7 x2	2 x1	7 x1, 3 x2	7 x1, 2 x1	7 x1	7 x4	7 x2	7 x1, 5 x1, 3 x1	3 x1, 7 x1
**156**	4 x1	4 x1	4 x1, 5 x1	7 x1, 5 x1	4 x1, 3 x1	2 x1	2 x2	3 x3	7 x4	NA	7 x1, 4 x1	4 x1	2 x2
**157**	6 x2	2 x1	6 x1	2 x1	2 x1	3 x1	7 x2, 6 x1	4 x1	7 x3	2 x1	6 x1, 2 x1	2 x1	NA
**158**	5 x1, 6 x1	6 x1, 7 x2	7 x1	7 x1, 6 x1	7 x3	7 x1	7 x3, 6 x1	4 x1	NA	2 x1	4 x1	2 x1	2 x1
**251**	NA	NA	4 x1	NA	1 x1, 6 x1, 7x1	NA	7 x2	NA	NA	2 x1	1 x1	1 x1	2 x1
**252**	NA	2 x1	4 x1	NA	1 x1	NA	NA	NA	NA	2 x1	1 x1	1 x1	2 x1
**253**	6 x1, 7 x1	7 x1, 3 x1	7 x1	2 x2	2 x1	3 x2	7 x1, 3 x2	2 x2	2 x2	2 x1	7 x3	2 x1	7 x3, 6 x1
**254**	6 x1, 4 x1	2 x2	2 x2	NA	2 x1, 3 x1	7 x1	7 x4, 3 x1	2 x2, 3 x1	6 x2	1 x2	7 x3, 4 x1	3 x2	6 x1, 3 x1
**255**	7 x2	2 x1	7 x1	3 x1, 6 x1	4 x1	3 x1	2 x1	3 x3	7 x1, 5 x1	5 x1	7 x3	4 x1	2 x2
**256**	7 x1, 6 x3	NA	6 x2	7 x2	4 x1, 6 x1, 7 x1	4 x1, 6 x1	7 x4	NA	7 x6	6 x1	6 x1, 5 x1	4 x1	6 x3
**257**	4 x2	2 x1	4 x1	2 x1	2 x1, 3 x1	2 x1, 3 x1	2 x2	3 x1	3 x1	2 x2	2 x1	2 x1	3 x1
**258**	7 x1, 6 x2	2 x1	NA	6 x1	NA	2 x1	2 x3	3 x1	2 x1, 4 x1	2 x1	2 x1, 3 x1	2 x1	3 x1, 6 x1
**351**	7 x4, 6 x1	NA	5 x1	2 x3	1 x1, 2 x1, 6 x1	2 x1	2 x1	2 x1, 3 x1	2 x1	3 x2	1 x2	2 x2	7 x4
**352**	7 x2, 5 x1	2 x1	7 x2, 4 x1	2 x1	1 x1, 3 x2	2 x1	3 x2	7 x1, 4 x1	7 x4	2 x1	1 x1, 2 x2	2 x1	3 x2
**353**	4 x1	2 x2	1 x1, 2 x1	2 x1	3 x1	3 x1, 7 x2	2 x1	2 x2	2 x2, 3 x1	NA	7 x3, 5 x1	NA	3 x1
**354**	6 x2	2 x2	3 x2, 4 x1	NA	7 x2, 6 x2	4 x1, 7 x1	7 x4	NA	7 x3	1 x1	7 x3, 3 x1	4 x1	7 x3, 6 x1
**355**	5 x1, 3 x1	6 x1	3 x1	6 x1	NA	2 x1	3 x2	4 x2	2 x1	3 x1	2 x2	2 x1	1 x2
**356**	3 x1	6 x1, 3 x1	2 x1	2 x1	NA	2 x1	2 x1	2 x2	2 x1	NA	7 x3	2 x1	7 x2
**357**	5 x1, 6 x1	2 x2	5 x1, 4 x1	2 x1	3 x1	NA	6 x1, 5 x1	NA	7 x4	NA	4 x2	4 x1, 7 x1	5 x1, 6 x1, 7 x1
**358**	3 x1	6 x1, 7 x1	2 x1	7 x1, 5 x1	7 x2, 5 x1	NA	4 x3	4 x2	3 x2	2 x1	2 x2	2 x1	2 x2
