# Supplementary material for: Effects of orally administered crofelemer on the incidence and severity of neratinib-induced diarrhea in female dogs
Source: PLoS One. 2024 Jan 24;19(1):e0282769. doi: 10.1371/journal.pone.0282769 (PMC10807780; doi:10.1371/journal.pone.0282769)
Supplement: S9 Table — (PDF) [file pone.0282769.s010.pdf]

S9 Table. Daily hydration scores by treatment group over the 4-week crofelemer study period in neratinib-induced diarrhea in dogs (n=8 per treatment group).

Hydration Score

Animal Number	Day 0	Day 1	Day 2	Day 3	Day 4	Day 5	Day 6	Day 7	Day 8	Day 9	Day 10	Day 11	Day 12	Day 13	Day 14	Day 15	Day 16	Day 17	Day 18	Day 19	Day 20	Day 21	Day 22	Day 23	Day 24	Day 25	Day 26	Day 27
**151**	E	E	E*	M	E	E	E*	E	E*	E	E	E	E	E	M*	E	E	E	E	E	E	E	E	E	E	E	E	E
**152**	E	E	E	E*	E	E	E	E	E	M	E	E	M*	O**	M*	M*	O*	E	M*	E	E	E	E	E	E	E	E	E
**153**	E	E*	E*	E	E	E	E	E	E	E	E	M*	M*	E	M*	E	M*	E	E	E	E	E	E	E	E	E	E	E
**154**	E	E	E	E	E	E	E	E	E*	O	E	E	E	E	E	E	E	E	E	E	E	E	E	E	E	E	E	E
**155**	E	E	E	E	E	E	E*	E*	E*	E	E	E*	E	M*	E	E	E	E	E	E	E	E	E	E	E	E	E	E
**156**	E	E	E	E	E	M*	M	M*	M*	M*	O*	E	M	E	O*	M*	M*	M*	M*	O*	E	E	E	E	E	E	E	E
**157**	E	E	E	E	E	E	M*	M*	M*	O*	O*	O*	M*	M*	E	E	E	E	E	E	E	E	E	E	E	E	E	O
**158**	E	E	E	E	E	E	E	E	E	E	E	E	E	E	E	E	E	E	E	E	E	E	E	E	E	E	E	E
**251**	E	E	E*	M*	E	E	E	E	E*	E	E	E	E**	M*	E	M*	E	E	E	E	E	E	E	E	E	E	E	E
**252**	E	E	E	E	E	E	E	E	E	E	E	E	M	M*	E*	E	E	M*	E	E	E	E	E	E	E	E	E	E
**253**	E	E*	M*	E	E	E	E	E*	E*	E*	E	M*	E*	E*	E	M	E	E	E	E*	E	E	E	E	E	E	E	E
**254**	E	E	E	E	E	E*	E	E*	E	E	M*	M*	M**	E*	E	E	E	E	E	E	E	E	E	E	E	E	E	E
**255**	E	E	E	E	E	E	E	M*	E	E	E	E*	E	M*	E	E	E	E	E	E	M*	E	E	E	E	E	E	E*
**256**	E	E	E*	E	E	M*	E	E	M*	M*	M*	E	E	E	E*	E	E	E	E	E	E	E	E	E	E	E	E	E
**257**	E	E	E	E	E	E*	E	E	E	E	E	E	E	E	E	E*	E	E	E	E	E	E	E	E	E	E	E	E
**258**	E	E	E	E	E	E	E	E	E	E	E	M*	E	E	E	E	M*	E	E	E	E	E	E	E	E	E	E	E
**351**	E	E	E	E	E	E	E	M*	E*	O*	M*	E	M*	M*	M*	E	E*	E	E	E	E	E	E	E	E	E	E	E
**352**	E	E	E*	E*	E	E	E*	E*	E*	E	E	E	E	E	M*	E	E	E	E	E	E	E	E	E	E	E	E	E
**353**	E	E	E	E	E	E	M*	E	E*	E	E	E	E	E	E	E	E	E	E	M*	E	E	E	E	E	E	E	E
**354**	E	E	E	E	E	E	E	E*	M*	E	M*	M**	O**	M	O*	M*	E	E	E*	E	E	E	E	E	E	E	E	E
**355**	E	E	E	E	E	E	E*	E*	E	E	E	E	E	E*	E	E	E	E	E	E	E	E	E	E	E	E	E	E
**356**	E	E	E	E	E	E	E*	E	E*	E	E	E	E	E	E	E	E	E	E	E	E	E	E	E	E	E	E	E
**357**	E	E	E	E	E	E	E	E	M*	E*	E	M*	M*	E	E	E	E	M*	M*	E	E	E	E	E	E	E	E	E
**358**	E	E	E	E	E	E*	M*	O*	E	O*	E	M*	O**	M*	M*	E	E	M*	M*	E	M*	E	E	M	E	E	E	E

E = Euhydrated; M = Mild Dehydration; O = Moderate Dehydration; S = Severe Dehydration; *=150 mL LRS given; **= 300 mL LRS given
