# Supplementary material for: Effects of orally administered crofelemer on the incidence and severity of neratinib-induced diarrhea in female dogs
Source: PLoS One. 2024 Jan 24;19(1):e0282769. doi: 10.1371/journal.pone.0282769 (PMC10807780; doi:10.1371/journal.pone.0282769)
Supplement: Attachment — Submitted filename: PONE-D-23-05162_reviewerComments.docx [file pone.0282769.s011.docx]

Comments for the Authors:

- The study presents the results of primary scientific research.
  - Yes
- Results reported have not been published elsewhere.
  - Yes: This appears to be the same study that was published in BioRx in 2023 by the same authors, although that is not a formally peer-reviewed journal, so would leave it up to the PlosONE editor if this is ok to publish in this journal: “Guy, Michael K., et al. "Effects of orally administered crofelemer on the incidence and severity of neratinib-induced diarrhea in female dogs." *bioRxiv* (2023): 2023-02.”
- Experiments, statistics, and other analyses are performed to a high technical standard and are described in sufficient detail.
  - No. Only several brief and “representative” examples of the raw data are submitted in this work. The majority of statistics appear to have been performed using arbitrary cut offs to define responders vs non-responders rather than using the raw stool scale consistency numbers. And there were no data or statistics presented that describe multiple components of the study include hydration status, oral intake, dose changes, subQ fluid administration.
- Conclusions are presented in an appropriate fashion and are supported by the data. The main conclusion that crofelemer prophylaxis can reduce the incidence of diarrhea in this animal model could be supported by the data. But my concern is that the data presented is the result of several pre-statistical calculations using arbitrary cut-offs to batch label animals into responder vs non-responder phenotypes rather than using raw data for definitive analysis. The statistics here appear biased towards the hypothesized effect.
- The article is presented in an intelligible fashion and is written in standard English.
  - Yes
- The research meets all applicable standards for the ethics of experimentation and research integrity.
  - Yes.
- The article adheres to appropriate reporting guidelines and community standards for data availability.
  - No. Multiple data sets are absent and intermittently referenced in the discussion. These include data from weeks 2 and 3, data on food and water intake, episodes of emesis, which animals required changes in dosing regimen, including the size of the dose change and the timing of the change. There are no lab data sets reported even though the authors note that these were obtained. And the authors make several references to intestinal histology obtained after animals were sacrificed that was unremarkable and normal but these data were not shown.
- Specific comments follow:
- Abstract
  - Would include confidence intervals if there’s room
  - Why were these dosing regimens chosen? A similar study was performed in patients receiving trastuzumab, pertuzumab, and taxane in the HALT-D trial and crofelemer was administered as 125mg PO BID
- Conflict of interest statement:
  - The authors report no competing interests, however the lead author appears to be employed by Napo Pharmaceuticals which manufactures crofelemer under the trade name Mytesi ®, the drug being interrogated in this study. Unclear if Napo Pharmaceuticals funded this privately or not and this should be noted, as the authors reported that this project was not specifically funded. This should be noted.
- Figure 1: Examples of non-responder and responder dogs
  - Figure is difficult to interpret and appears low-quality, can this be converted to a graph? Are these respresentative of two single dogs? Why not show all the data?
- Figure 2: Percentage of dogs by treatment group that were determined to be responder dogs over the entire 4-week study period of crofelemer prophylaxis in neratinib-induced diarrhea in dogs
  - Recommend showing all data points on these graphs, would also define what is considered a responder in this graph. Further, categorizing animals as a responder vs non-responder despite still having loose and frequent stools is suggestive of some underlying bias, would consider graphing the raw data (e.g., graphs of loose stool number and stool consistency), also there are no statistics demonstrated on this graph, is this statistically significant?
- Figure 3: Average number of watery stools by treatment group over the entire 4-week study period of crofelemer prophylaxis in neratinib-induced diarrhea in dogs.
  - This graph lacks and needs statistics, are these not significantly different? Why is the graph changes to a horizontal rather than vertical? Would keep the order of the groups the same (figure 2 is CTR, QID, and then BID; while this graph is BID, QID, CTR)
- Table 1: Average number of water stools per week by treatment group over the 4-week crofelemer study period in neratinib-induced diarrhea in dogs (n=8 per group).
  - Would define “WS” in the figure legend, assuming it means “watery stools”, would also consider unifying the verbiage here since both WS and loose stools are used and appear to mean the same in this work, but if this is meant to convey a difference in severity, would state that more clearly; would keep group orders consistent between figures and tables; this table is useful for looking at the data, but a graph of this data should also be shown with ANOVA statistics comparing all groups, I’m assuming the p-values represent significant difference from CTR groups, but it is important to see whether or not there is a statistically significant difference between the experimental groups as well, which is not shown here, although clearly not significant
  - Further, why were weeks 1 and 4 chosen, what happened in weeks 2 and 3?
  - I found the footnote helpful to understand this table, might include statistics down there as well
- Table 2: Average number of loose/watery stools per week per neratinib dose by treatment group over the 4-week crofelemer study period in neratinib-induced diarrhea in dogs (n = 8 per group).
  - Here “loose/watery” is used, would be consistent throughout the work
  - I don’t feel that this figure adds too much, since all animals received the same regimen of neratinib, and only the first 5d used daily 40mg dosing and the following 23 days used 80mg once daily dosing, if there is a difference in stool output, would like to see some statistics comparing the loose stools per dose between the groups (consider a nested anova)
- Table 3: Average purina fecal scores (PFS) for stool consistency per week by treatment group over the 4-week crofelemer study period in neratinib-induced diarrhea in dogs (n=8 per group).
  - I am not familiar with the PFS system, would provide a key to accompany this table, a quick google search shows that it appears to be similar to the Bristol stool scale used in humans
  - Use consistent table order as recommended above
  - Where is the data from Weeks 2 and 3?
  - What is the difference between this table and table 1? This table seems to be more objective with use of the PFS scale
  - Recommend showing statistics between all groups with nested anovas, are QID and BID significantly different from each other? I’m assuming not
  - Also, for all p-values in these tables, would make it more clear that they are comparing experimental to control groups
- Table 4: Trend of number of weekly dose reduction of neratinib by treatment group over the 4-week crofelemer study period in neratinib-induced diarrhea in dogs (n = 8 per group)
  - Where is week 2-3 data?
  - How were the doses reduced and what was the criteria for reduction?
- Materias and Methods
  - Animals
    - Why is randomization important here? Is this specifically due to differences in weight and age?
  - Study design
    - Why were these doses chosen? Are neratinib or/and crofelemer weight based? If so, these doses may lead to significantly higher drug concentrations than those used in patients undergoing treatment with neratinib or/and crofelemer since 125mg BID seems to be the current recommended dosing regimen for the 70kg human
    - If these tablets are delayed-release, why is the QID regimen required? What is the half-life of this formulation and average therapeutic window?
    - Further, it is noted in the table footnotes and in a later methods section that the dose of neratinib was increased to 80mg daily at day 6-23 due to emesis in the first few days, would include that in the study design portion for clarity
    - The sample size seems adequate to evaluate an effect against placebo, which confounders that were not controlled are being suggested here that are due to sample size?
  - Housing and Husbandry
    - Is the 30-70% humidity range standard?
    - Were the intake volumes of food and water recorded and quantified by individual animals in this study? It would be good to know that all animals had approximately the same intake of food and water throughout this diarrhea study, especially since the neratinib dose is increased at day 6.
  - Dose Administration, Animal Care, and Monitoring
    - It would be interesting to see the emesis data here, as well as a graph showing which doses were admitted from which animals for clarity; for example, did the dose or frequency of crofelemer affect emesis at all?
    - What is meant by “neratinib was titrated daily”? in the study design, it is referenced as either 40mg or 80mg, this needs to be described and the data needs to be shown in better detail to improve the rigor of this work
    - Further, there did not appear to be any tables or graphs comparing the symptoms of anorexia, lethargy, dehydration, vomiting, or bloody diarrhea, this should be included as part of therapeutic and adverse event monitoring
  - Data collection
    - Would include body weight, subQ fluid admin, and food data in this work rather than only referencing that it was recorded
    - Fecal score was collected twice daily in this study, is this an aggregate of several bowel movements? Would consider including the mass or volume of the stools in these collections as part of the quantitative data. How do the animals defecate? Through the cages, is there a designated area?
  - Ethical Statement
    - It is noted that blood was drawn for clinical pathology but this data was not presented, would include if possible, as well as any tissue data that was collected (for example, any evaluation of intestinal epithelium between the groups?)
    - Would consider referencing standard euthanasia protocol here
  - Statistical Methods
    - It is noted that no data was excluded, but data from Weeks 2 and 3 are generally missing throughout this work, would include these

It is noted that PFS of 6 or 7 was considered watery bowel movements and anything <6 was considered normal. However, according to the published PFS, only 7 should be considered watery, 6 would be considered “has texture, but not defined shape; occurs as piles or as spots; leaves residue when picked up”; and would use caution with calling 1 or 3-5 as “normal” as these are not described as normal in this published literature. Would consider not grouping PFS numbers together and simply quantify the raw data. This could be done in a scatter plot format, with each experimental group represented by a different line, similar to what has been shown for murine norovirus (copied here, “Roth, Alexa N., et al. "Norovirus infection causes acute self-resolving diarrhea in wild-type neonatal mice." *Nature Communications* 11.1 (2020): 2968.”):

- I would caution against displaying this data as responder vs non-responder and would base an effect more on the stool consistency as an objective measure
  - Why was <7 watery bowel movements per week chosen for the responders? What is the average normal expected watery bowel movement for this animal model?
  - How was the dose of neratinib required to maintain a consistent level of diarrhea? Was this determined in the study or was there a preliminary study used to determine this? If the dose of neratinib had to be altered to maintain a diarrheal phenotype in the placebo, than that would argue against the utility of crofelemer during neratinib treatment since animals receiving placebo adapted to neratinib w/o specific intervention. It is not enough to say that subQ fluid admin and dose changes of neratinib were analyzed using similar procedures, since these two aspects alone could entirely explain any alterations in fluid-content of stools in this study. This data needs to be shown, explained, and statistics analyzed in a dedicated figure or table.
  - Is the effect size in this study 1.0? If so, the study may be underpowered, but that does appear to be noted elsewhere.
- Results (reviewer received a non-numbered article)
  - First sentence: authors describe there were no deleterious effects on the dog’s behavior, however in the methods they noted that some animals had emesis requiring modifications to the study, would describe that more consistently
  - I like that the animal and human dose comparison was made, would include in the methods section, however a quick google search shows that common starting regimens of neratinib is 240mg once daily for a year which is ~3.4mg/kg for a 70kg human, would clarify. There is also some reference to even less in the starting dose, around 120mg daily with a slow up-titration.
  - See figure comments for specific comments related to the data
  - Would simply describe the raw data using the stool scale numbers, my concern is that the effect of responder vs non-responder may be overstating the effect when a more careful analysis of the PFS numbers would reveal more subtle changes in stool consistency that would be important points to make when translating this work to patients who are often on multiple anti-diarrheals. For example, in clinic, we don’t say “responder” or “non-responder”, we like to quantify the stool number and consistency, and characterize the stool as bloody vs non-bloody, along with other symptoms such as abdominal pain, etc. Inclusion of this data would improve the rigor of this work.
  - Week 2 and 3 data is not mentioned aside from overall data, is there an expectation for delayed response to crofelemer?
  - Would mention if there is a statistically significant difference between treatment groups, rather than only compared to placebo group
  - Paragraph that references table 3: the first sentence doesn’t make sense, would check for accuracy. What is meant by “significantly greater stools”? I like that the PFS are reported here, would also show daily trends per group. It is also important to note any changes to dosing regimens made to maintain diarrheal phenotype as briefly described in the methods. Some grammar corrections need to be made.
  - The data in table 4 does not appear to be statistically significant in the current format, would consider showing the individual animal data points here rather than averages. Table 4 does not describe subQ fluids, would include that data.
  - I like that the path was described in the final section, but would include the H&E of the distal ileum and colonic mucosa at the very least as a figure as well. There is also evidence in the literature for histopathologic changes in distal ileal and colonic mucosa following 2-4 weeks of neratinib in other animal models, would contrast your findings with those of prior work and justify why the results are different (different animal model vs dosing regimen, etc).
- Introduction
  - References are inconsistent in the sentences, would fix in final copy-editing
  - I like this introductory paragraph, but would add a sentence of the mechanism by which TKIs lead to diarrhea going beyond simply stating that they target EGFR, TKs, and HER2--are there changes in ion transport, inflammation with decreased intestinal barrier integrity? Etc
  - Would add reference for function of crofelemer, also an earlier description of the ion-transport basis of diarrhea would clarify why the authors chose this chloride-ion transporter inhibitor to study
  - What is the actual mechanism by which crofelemer inhibits Cl- transport? Does it sterically block these channels?
- Discussion
  - Nice background on mechanism underlying crofelemer, would put this in the intro
  - Recommend showing the data with regards to body weight, hydration status, food consumption, and “other hematological or clinical chemistry parameters”
  - It is notable that the authors state the absence of histopathologic changes in intestinal tissues was consistent to the findings of that in a human study (Liu et al 2022), when in that study, only ~7% of patients receiving TKI therapy underwent colonoscopies, with ~50% showing no path, ~33% with non-ulcerative inflammation, and the rest with ulcers. Thus the results in this work, which are only stated and not shown, are not consistent with this prior study.
  - New data is being presented in the discussion with a 6% neratinib dose increase in crofelemer treated animals and a %% reduction in subQ fluid replacement. New data should not be presented in the discussion, and this data should have adequate graphical or tabular descriptions with appropriate statistical analysis. Further, 6% dose increases were not noted in the study design, it is unclear why this was done.
  - Throughout the discussion and this work, multiple different stool consistency grading mechanisms are introduced without description or an example chart, would add either a description or a chart for understanding
  - The loperamide trials in the discussion would be more appropriate in the introduction as a reason why we need new and improved anti-diarrheals, but definitely an important point to make
  - Authors again reference that there are no histopathologic findings in prior studies when there actually were both macroscopic and microscopic findings in citations 14, 16, and 17. Of note, citation 17 is a case report of a patient with UC, status-post colectomy, who presented with duodenitis that was successfully treated with tacrolimus and does not appear to be relevant to this work
  - The crofelemer background is repeated in the discussion, can take this out, but would also describe the half-life and bioavailability in more detail given the frequent (QID) dosing regimen used in this study. This could explain the lack of difference between BID and QID groups
- Acknowledgements
  - The authors report that there are no financial or non-financial conflicts of interest as all authors are employees or consultants of Napo Pharmaceuticals, Inc. Which is actually a notable COI, because it is this company that markets crofelemer.
