# Supplementary material for: Effects of orally administered crofelemer on the incidence and severity of neratinib-induced diarrhea in female dogs
Source: PLoS One. 2024 Jan 24;19(1):e0282769. doi: 10.1371/journal.pone.0282769 (PMC10807780; doi:10.1371/journal.pone.0282769)
Supplement: Attachment — Submitted filename: PONE-D-23-05162_Response to Reviewers.docx [file pone.0282769.s012.docx]

Responses to reviewers from the authors were prepared on October 16^th^, 2023.

As requested, the Supplemental Figures and Tables have been removed from the manuscript and have been submitted in a separate file titled “Supporting Information’.

Also, the legend for each item in the ‘Supporting Information’ file has been listed in the manuscript after the references list and is listed below:

**Supplemental Information**

Supplemental Figure S1. Scoring chart for the Purina Fecal Scoring (PFS) System

Supplemental Table S1. Body weight at enrollment and body weight changes per week by treatment group over the 4-week crofelemer study period in neratinib-induced diarrhea in dogs (n=8 per treatment group).

Supplemental Table S2. Food consumption per week by treatment group over the 4-week crofelemer study period in neratinib-induced diarrhea in dogs (n=8 per treatment group).

Supplemental Table S3. Weekly average volume (mL) of subcutaneous fluid administration each week by treatment group over the 4-week crofelemer study period in neratinib-induced diarrhea in dogs (n=8 per treatment group).

Supplemental Table S4. Adverse event profile from Days 0 through 28 of 4-week crofelemer study period in neratinib-induced diarrhea in dogs (n=8 per treatment group.

Supplemental Table S5. Number of dogs with out of range clinical chemistry and hematology parameters on study Days 0, 7, 14, 21, and 28 by treatment group over the 4-week crofelemer study period in neratinib-induced diarrhea in dogs (n=8 per treatment group).
